# Effectiveness of Gamified Teaching in Disaster Nursing Education for Health Care Workers: Systematic Review

**DOI:** 10.2196/74955

**Published:** 2025-07-09

**Authors:** Shiyi Bai, HuiJuan Zeng, Qianmei Zhong, Lulu Cao, Mei He

**Affiliations:** 1 Mianyang Central Hospital, School of Medicine, University of Electronic Science and Technology of China, No.12 Changjiaxiang, Mianyang, 621099, China, 86 13778440262

**Keywords:** gamification, health care workers, disaster nursing, effectiveness, systematic review, PRISMA

## Abstract

**Background:**

With the continuous advancement of medical technology and the frequent occurrence of disaster events, the training of health care workers in disaster nursing has become increasingly significant. However, traditional training methods often struggle to engage learners’ interest and enthusiasm, making it challenging to simulate emergencies in real-life scenarios effectively. Gamification, as an innovative pedagogical approach that enhances the enjoyment and practicality of learning through the incorporation of game elements, has garnered considerable attention in the realm of disaster nursing education for health care workers in recent years. This review systematically evaluates its effectiveness and explores its advantages in improving training outcomes.

**Objective:**

This review aims to evaluate the effectiveness of gamified teaching methodologies in disaster nursing education and to identify the outcome of 16 indicators used in existing studies.

**Methods:**

This study was conducted following the PRISMA (Preferred Reporting Items for Systematic reviews and Meta-Analyses) guidelines, using the PICO-SD framework (Population, Intervention, Control, Outcome, Study Design) to establish the inclusion criteria. The researchers systematically searched 8 databases on February 10, 2025, including the Cochrane Library, PubMed, CINAHL (EBSCO), Embase, Web of Science, CNKI, Wanfang, and SCOPUS. Ultimately, 16 quasi-experimental studies investigating the application of gamified teaching in disaster nursing education were included in the analysis. For randomized controlled trials (RCTs), the Cochrane Risk of Bias Assessment Tool (RoB 2.0) was used for quality assessment; for quasi-experimental studies, the Joanna Briggs Institute Risk of Bias Tool for Non-Randomized Intervention Studies was used for methodological quality evaluation. Given the heterogeneity of study designs and the diversity of study indicators, this study used a narrative synthesis to integrate the findings.

**Results:**

The studies included in this review comprised 1 RCT and 15 quasi-experimental designs. Six gamified formats exhibited positive outcomes. The effectiveness of these formats was assessed through various metrics, including theoretical knowledge (14 studies), practical skills (11 studies), learner satisfaction (9 studies), knowledge retention (4 studies), and self-efficacy (2 studies). All formats demonstrated improvements in knowledge and skills, with high levels of satisfaction reported. However, data on long-term retention were limited.

**Conclusions:**

Gamification teaching methods have shown significant potential to enhance core competencies such as emergency response, decision-making, and teamwork in disaster nursing education and have been effective in reinforcing learning engagement through elements such as cooperation, competition, scoring, and scenario simulation. However, there is a lack of standardized assessment frameworks for skill acquisition, longitudinal studies tracking behavior in real-life scenarios, and rigorous RCTs comparing it with traditional instruction. Although the existing evidence has not systematically confirmed its full effectiveness, based on the findings, this paper provides practical recommendations for developing and implementing gamified teaching strategies in disaster nursing education to enhance students’ knowledge acquisition and practice.

## Introduction

According to the Global Disaster Data Platform, it is projected that in 2024, approximately 20.83 million disaster events will occur globally, affecting a cumulative total of 242,061,500 individuals and resulting in direct economic losses estimated at around US $240.1 billion [1]. The frequency of both natural and anthropogenic disasters, including floods, major accidents, and terrorist attacks, not only causes significant human casualties and psychosocial trauma but also leads to systematic infrastructure degradation [2]. Such events are often abrupt and unpredictable, with the extent of destruction frequently exceeding the capacity and resource allocation of local emergency response systems, thereby posing substantial challenges to global sustainable development goals [3,4]. In this context, enhancing disaster response capabilities has transitioned from a selective preparedness option to a mandatory requirement within the health care sector [5].

As the primary human resource within the health care system, nursing workers play crucial roles in casualty classification, emergency treatment, and public health management during disaster relief efforts [6]. This necessitates that practitioners not only possess a robust foundation of medical knowledge but also demonstrate the practical ability to make rapid decisions and execute precise rescue operations in complex environments [7]. However, significant limitations exist within the current framework of disaster care education: the traditional pedagogical approach, which emphasizes knowledge transmission, inadequately simulates the dynamic complexities of disaster scenarios. This results in notable deficiencies in knowledge transfer and the development of situational resilience [8], proving insufficient to meet the rapidly escalating demand for disaster relief training [9].

In this context, gamification—defined as the integration of game mechanics such as points, narratives, and instant feedback into educational scenarios—demonstrates significant advantages. Education research indicates that this model can markedly enhance learners’ motivation and improve knowledge retention by promoting interactivity, challenge, and immersion [10-12]. In health care disaster education, gamified instruction has demonstrated efficacy in cultivating time-sensitive triage decision-making and optimizing cross-functional collaboration under resource constraints [13,14]. Recent studies have shown that simulating mass casualty events in tabletop games can significantly improve knowledge recall rates. The experimental group achieved long-term memory levels of up to 97% without additional learning interventions. Meanwhile, students had a very positive attitude toward this educational model [15]. There is a growing international consensus that essential game elements, including achievement systems, progressive challenges, and contextualized feedback, effectively facilitate the translational application of complex nursing skills [16,17].

It is important to emphasize that although the effectiveness of gamified teaching in nursing education has been preliminarily validated [18], its implementation in disaster nursing education for health care workers is still in the exploratory stage, and its effectiveness and best practice models have not yet been fully validated. Therefore, this study will investigate the effectiveness of gamified teaching interventions in disaster nursing education and analyze the indicators to assess their effectiveness, aiming to guide instructional design and practice in related fields.

## Methods

### Design

The quantitative studies were systematically evaluated following the Cochrane Handbook for Systematic Reviews of Interventions, and the report adhered to the PRISMA (Preferred Reporting Items for Systematic Reviews and Meta-Analyses) guidelines [19]. The study protocol was preregistered on PROSPERO (CRD420250652129) [20]. Ethical approval and informed consent were not necessary, as this study did not include individual patient data.

### Literature Search

This study systematically searched 8 major electronic databases, including Cochrane Library, PubMed, CINAHL (EBSCO), Embase, Web of Science, CNKI, Wanfang, and SCOPUS. The search strategy was refined with the assistance of health information librarians, using Boolean logic operators to integrate index terms (including MeSH, Emtree, and CINAHL subject terms) with free text terms. The complete search strategy is detailed in Multimedia Appendix 1. To prevent excessively narrowing the search scope, outcome indicators were excluded from the selection criteria for search terms. The search period was confined to the time frame from database inception to February 10, 2025, and the literature was restricted to English and Chinese. Additionally, the search was augmented by manually screening references from included studies and pertinent review literature.

### Inclusion and Exclusion Criteria

To establish the framework for inclusion criteria, we used the PICO-SD model for this systematic review. The components of the study were as follows: (1) Population: nursing students or registered nurses; (2) Intervention: educational programs incorporating core gamification elements (eg, themed games, virtual reality simulations, tabletop games, escape rooms, serious games, etc); (3) Comparison: traditional teaching methods (theoretical lectures) and nongamified e-learning; (4) Outcome: knowledge acquisition, skill performance, learner satisfaction, and evaluation of teaching effectiveness; and (5) Study design: randomized controlled trials (RCTs), nonequivalent control group designs, and single-group pretest and posttest studies. Exclusion criteria comprised (1) editorials, letters, conference abstracts, dissertations, and gray literature; (2) studies focused solely on virtual simulation that lacked game mechanics; (3) mixed-sample studies involving other health care professionals or nonnursing professionals; and (4) nonempirical research designs (eg, theoretical explorations and protocol designs).

### Quality Evaluation

The quality of studies included in RCTs was evaluated using the Risk of Bias Assessment Tool for Randomized Controlled Trials developed by the Cochrane Collaboration [21]. Conversely, non-RCTs (quasi-experimental studies) were assessed for methodological quality using the Joanna Briggs Institute tool for quasi-experimental studies [22]. The literature quality was independently evaluated for each project by 2 systematically trained researchers (SB and HZ), with any discrepancies resolved by a third reviewer.

### Data Extraction

All retrieved documents were imported into EndNote 20 for deduplication and classification management [23]. A structured data extraction form, tailored to meet the study objectives, was developed through discussions among the research team and included the following core elements: authors/year, country, study design, population and sample size, intervention, comparison, outcome, and key findings. The literature-screening process was conducted in 2 stages: initially, 2 uniformly trained researchers (SB and HZ) independently performed the initial title and abstract screening, followed by a full-text rescreening of potentially eligible literature. Any disagreements were resolved through third-party arbitration to reach a consensus. The final data extraction was carried out by 1 researcher (SB) and subsequently cross-checked by the other 2 members to ensure accuracy.

### Data Synthesis

Following the search and data screening, we will organize and summarize the research data in a tabular format. First, due to the diversity of research design components and outcome measures, meta-analysis was deemed unfeasible. Therefore, we used Campbell’s narrative synthesis (synthesis without meta-analysis) to integrate the evidence [24]. Second, the extracted data were analyzed to assess the effectiveness of interventions using various types of games, aiming to ascertain whether gamified instruction positively influenced outcomes. Finally, the extracted data were examined for indicators of effectiveness, summarizing the categories of effectiveness indicators and offering recommendations for the future implementation of gamification in disaster nursing education.

## Results

### Selection Process

**Figure 1.**
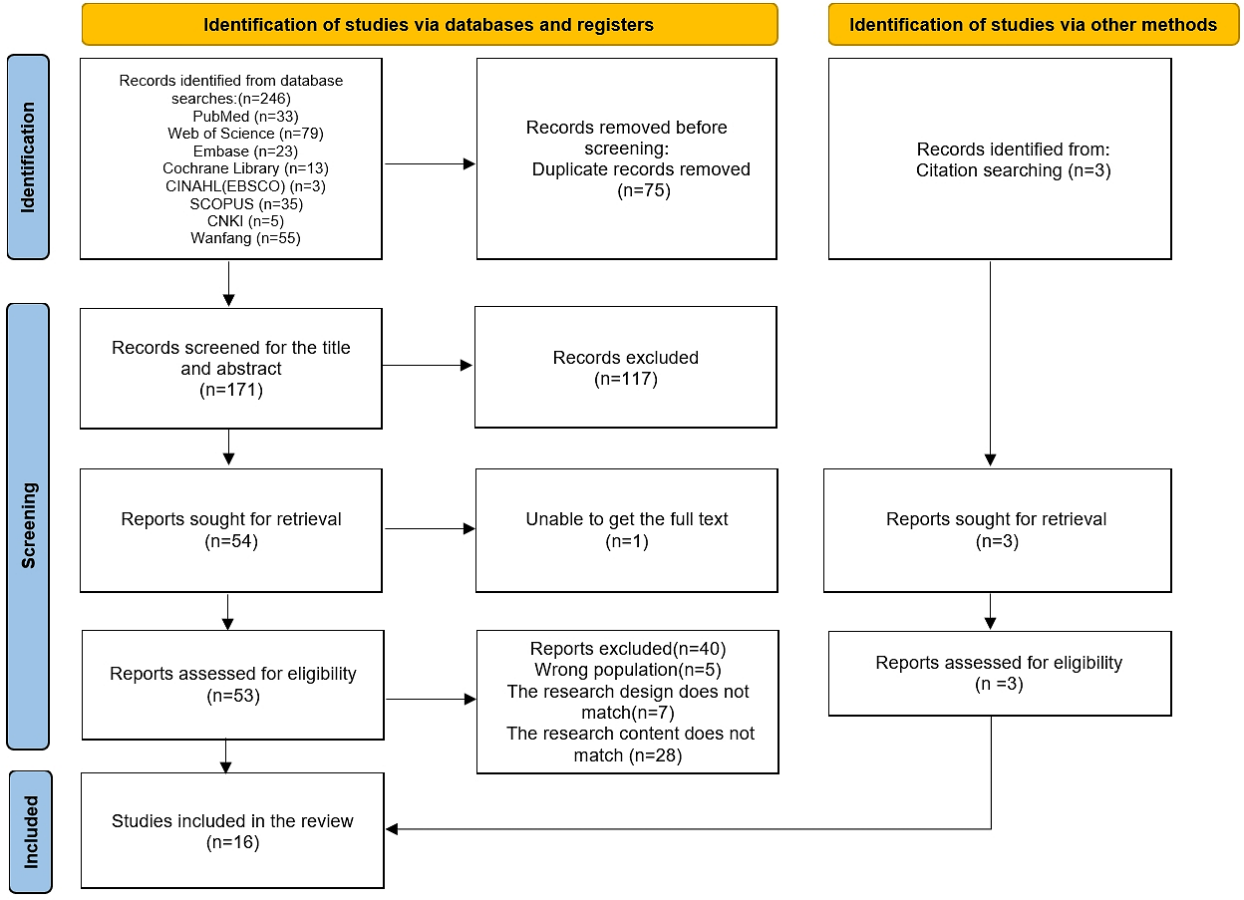
Flowchart of literature screening.

Following the initial screening, 246 studies were identified, of which 171 remained after deweighting. A further 117 were excluded based on the title and abstract review, leaving 54 studies. Subsequent full-text screening led to the exclusion of 40 studies due to inaccessibility, incomplete information, or irrelevance to the topic. Additionally, 3 studies were sourced from the references, culminating in the inclusion of 16 studies [19]. The modified PRISMA flowchart (Figure 1) delineates the comprehensive study selection process.

### Study Characteristics

This systematic review encompasses a total of 16 research studies, published between 2010 and 2024. The fundamental characteristics of the included literature are shown in Table 1.

**Table 1.** Basic characteristics of included studies.

Authors/year/country	Study design	Population and sample size	Intervention			Key finding
			Game category	Gamification elements	Outcome	
Moon and Kim (2024, Korea) [25][Bibr R25]	Quasi-experimental design—single group pre- and posttest	Emergency department nurses (n=27)	VR-MGBA^a^ (100 minutes)	Points Ranking Feedback Scenario-based exercises	Critical thinking Triage skills Triage accuracy App usage details	Gamification improves emergency triage accuracy.
Wang et al (2024, China) [26][Bibr R26]	Quasi-experimental design—single group pre- and posttest	Emergency medical nurses (n=97)	Tabletop games (3 hours)	Challenges Collaboration Feedback Map	Knowledge Attitude Feedback	Improved triage skills and motivation.
Cha and Lee (2023, Korea) [Bibr R27][27]	Quasi-experimental design—single group pre- and posttest	Clinical nurses (n=30)	Tabletop games (60 minutes)	Scenario-based exercises	Knowledge	Effective in improving knowledge of emerging infectious diseases.
Masoumian Hosseini et al (2023, Iran) [Bibr R28][28]	Quasi-experimental design—2 groups of pre- and posttests	Nursing students (^b^*I*=60, ^c^*C*=60)	Simulation games (2 weeks)	Competitive incentives Scenario-based exercises Feedback	Skills Knowledge OSCE^d^	Enhances learning sustainability when integrated with instructional practices.
Hu et al (2022, China) [Bibr R29][29]	Quasi-experimental design—2 pre- and posttests, final test	Nursing students (*I*=82, *C*=85)	VR-MGBA (4 hours)	Scenario-based exercises	Knowledge Skills Satisfaction Engagement and knowledge retention	More effective than traditional lecture-based instruction.
Hu et al (2022, China) [Bibr R30][30]	Quasi-experimental design—2 pre- and posttests, final test	Nursing students (*I*=63, *C*=68)	VR-MGBA (6 hours)	Collaboration Scenario-based exercises	Knowledge Satisfaction Engagement and knowledge retention	Effective pedagogical tool for managing patient surges.
Masoumian Hossein et al (2022, Iran) [Bibr R31][31]	Quasi-experimental design—2 groups of pre- and posttests	Nursing students (*I*=30, *C*=30)	Theme games (4 weeks)	Collaboration Scenario-based exercises Competition Time pressure Feedback	OSCE Knowledge engagement and knowledge retention	Enhanced knowledge and behavioral fluency in crisis management.
Calik et al (2022, Turkey) [Bibr R32][32]	Quasi-experimental design—single-group pre- and posttest	Nursing students (n=62)	Serious games 40 minutes (1 week)	Scenario-based exercises	Knowledge Feedback	Increased knowledge of infections and safe behaviors.
Chow et al (2022, China) [Bibr R33][33]	Quasi-experimental design—single-group pre- and posttest	Nursing students (n=177)	3D games (75 minutes)	Scenario-based exercises Feedback Competition	Knowledge Self-efficacy	A valuable tool for managing low-probability clinical tasks.
Chang et al (2022, China) [Bibr R34][34]	Quasi-experimental design—2 sets of repeated measurements	Emergency nurses (*I*=32, *C*=35)	Simulation games (40 minutes)	Tabletop games Role-playing Feedback	Knowledge Self-efficacy Engagement and knowledge retention	Quickly boosts chemical disaster preparedness.
Ma et al (2021, China) [Bibr R35][35]	RCTs^e^	Nursing students (*I*=51, *C*=53)	Theme games (110 minutes)	Scenario-based exercises Cooperation Time pressure Feedback	Cognitive skills Affective	More effective than scenario simulation in improving disaster nursing competence.
Mirzaei et al (2020, Iran) [Bibr R36][36]	Quasi-experimental study—2 pre- and posttests	Clinical nurses (*I*=40, *C*=40)	Tabletop games (2 hours)	Scenario-based exercises	Knowledge Attitude Performance Knowledge retention	Improves nurses’ disaster preparedness.
Husna et al (2020, Indonesia) [Bibr R37][37]	Quasi-experimental study—single-group pre- and posttest	Nursing students (n=80)	Tabletop games (90 minutes)	Scenario-based exercises Role-playing	Knowledge Attitude	Enhances knowledge and attitudes toward learning.
Fathoni et al (2019, Indonesia) [Bibr R38][38]	Quasi-experimental study—2 pre- and posttests	Nursing students (*I*=18, *C*=18)	Tabletop games	Scenario-based exercises Role-playing	Knowledge	Significantly improves students’ knowledge.
Zhixia et al (2017, China) [Bibr R39][39]	Quasi-experimental study—2 pre- and posttests	Nursing students (*I*=166, *C*=165)	3D games	Scenario-based exercises	Knowledge Skills OSCE	Enhances students’ theoretical performance.
Knight et al (2010, United Kingdom) [Bibr R40][40]	Quasi-experimental study	Emergency nurses (*I*=47, *C*=44)	Serious gaming (60 minutes)	Scenario-based exercises Time-limited	Skills	Enhances learning and performance beyond traditional methods.

a VR-MGBA: virtual reality mobile game–based app.

b I: intervention group.

c C: control group.

d OSCE: Objective Structured Clinical Examination.

e RCTs: randomized controlled trials.

Among the 16 studies, geographical distribution reveals that 7 originated from China [26,29,30,33-35,38,39], 3 from Iran [28,31,36], 2 from Korea [25,27], 2 from Indonesia [37,38], 1 from Turkey [32], and 1 from the United Kingdom [40]. Notably, 93.75% (15/16) of the studies were conducted in Asian countries, highlighting the region’s active engagement in gamified teaching and learning. Regarding temporal distribution, only 2 studies were published between 2010 and 2018, while the number surged to 14 post-2019, indicating a significant increase in interest in gamified teaching within disaster nursing education. The study population comprised nursing students (n=1268) and registered nurses (n=392), with participant numbers per study ranging from 27 to 331, totaling 1660 participants in this systematic evaluation. Concerning game types, 6 categories of games were used in disaster nursing education, either in the intervention group or the control group. Specifically, 5 studies used tabletop games [26,27,36-38], 2 used serious games [32,40], 3 incorporated virtual reality mobile game−based app [25,29,30], 2 used featured theme games [31,35], 2 involved 3D games [33,39], and the remaining 2 used simulation games (interactive/VR) [28,34]. In terms of technological trends, upper-middle-income countries demonstrate a preference for integrating VR/3D technology with game elements for educational purposes, whereas low-income countries tend to favor low-cost tabletop games.

Furthermore, among these 16 studies, 6 used a quasi-experimental 1-group pretest and posttest design [25-27,32,33,37], 9 used a quasi-experimental 2-group pretest and posttest design [28-31,34,36,38-40], 1 study implemented an RCT [35], and 2 conducted various comparative analyses between games [34,35].

### Effectiveness of the Intervention

Research has shown that gamified instructional interventions exhibit differentiated characteristics in different disaster education scenarios. Tabletop games can significantly increase participants’ motivation, infectious disease awareness, and disaster care knowledge by simulating mass casualty events, infectious diseases, and natural disasters [26,27,36]. Serious games focus on reinforcing the theoretical system of disaster response capabilities by targeting the prevention of infectious diseases and the teaching of emergency triage [32,40]. Virtual reality mobile game−based app optimizes disaster evacuation training by relying on virtual reality technology to effectively improve triage accuracy, disaster classification knowledge, and student satisfaction [25,29,30]. The thematic game focuses on crisis management and disaster response capacity development, enhancing behavioral fluency and disaster care knowledge application through knowledge-behavior transformation mechanisms [31,35]. The 3D game is based on high simulation of mass casualty and trauma scenarios, which improves ease of use and extends the knowledge dimension of disaster relief [33,39]. Simulation games (interactive or virtual reality) are used in complex scenarios such as disaster triage and chemical disasters to strengthen skills, self-efficacy, and triage knowledge through immersive training, highlighting the synergistic optimization of practical ability and psychological state empowered by technology [28,34]. Table 2 summarizes the intervention effects of different types of games across various application scenarios.

**Table 2.** Intervention effects of different games.

Classification	Application scenario	Effectiveness of the intervention	Challenges
Tabletop games	Mass casualty incidents [26] Infectious diseases [27] Natural disasters [35] Disaster drills [36,37]	Participants’ motivation to learn is fostered Improved knowledge of infectious diseases Increased preparedness for natural disasters Improved disaster care knowledge and disaster response capacity	Need to coordinate multiple face-to-face participants. Reliance on manual feedback and recording.
Serious games	Prevention of infectious diseases [31] Teaching of critical incident triage [39]	Increased theoretical knowledge of disaster care capacity	High technical complexity (requires IT support). Interdisciplinary design team requirements.
VR-MGBA^a^	Disaster evacuation [25,29,30]	Increased accuracy of triage Increased theoretical knowledge of disaster classification Increased satisfaction	Device penetration limitations. Need for stability.
Theme games	Crisis and disaster management [31] Disaster care capacity [34]	Increased knowledge and behavioral fluency in crisis management Disaster care knowledge enhancement	Universal rate of equipment.
3D games	Mass casualty sites [33] Trauma scene [39]	Increased perceived ease of use of the game Increased knowledge of disaster response	Hardware compatibility issues.
Simulation games	Disaster site triage [28] Chemical disasters [34]	Increased disaster preparedness and self-efficacy Increased knowledge of disaster triage Skill-level improvement	Difficulty of algorithms for dynamic scene generation. Difficulty integrating with traditional curriculum.

a VR-MGBA: virtual reality mobile game–based app.

### Indicators of Effectiveness in Reporting Outcomes

The purpose of this study was to highlight the impact of interventions and assess their effectiveness. The 16 studies included in this review assessed the effectiveness of gamified instruction through a multidimensional perspective, focusing on key indicators such as theoretical performance, skill acquisition, participant satisfaction, long-term knowledge retention, and self-efficacy.

#### Theoretical Knowledge

In the 16 included studies, 13 (81.3%) assessed the intervention effect of gamified instruction on theoretical knowledge through systematic measurement. The measurements were mainly based on a multistage test design, including a pretraining test, a posttest, and a final test. For example, Moon and Kim [25] found a significant improvement in participants’ triage accuracy from a baseline level of 4.3 (SD 2.00) to 5.33 (SD 1.47) (*t*_27_=−2.18; *P*=.04) and a significant reduction in overtriage (*t*_27_=3.11; *P*=.00) by using a paired-sample 2-tailed *t* test. Mirzaei et al [36] used repeated-measures ANOVA with paired 2-tailed *t* tests to assess disaster care knowledge and showed that the intervention group’s disaster preparedness in terms of knowledge base, learning efficacy, and self-efficacy was significantly higher at posttest and 1-month follow-up period. A study by Chang et al [34] further demonstrated that the intervention group had a significantly higher self-assessment score of knowledge about chemical disasters than the control group (*P*<.05), but this difference disappeared at 3 weeks postintervention (*P*>.05), suggesting a limitation in the timeliness of knowledge retention.

#### Practical Skills

Of the 16 included studies, 7 (43.8%) assessed skill acquisition, of which 3 (18.8% of the total) used the Objective Structured Clinical Examination (OSCE) to quantify the effect of skill acquisition [28,31,39]. Masoumian et al [31] assessed the intervention effect of a thematic game on nursing students’ knowledge and behavioral fluency in crisis management through repeated-measures ANOVA and paired-samples 2-tailed *t* test. The study used a standardized rating scale in which the trainer quantitatively rated the participants’ skill performance in the patient assessment component of the theme game. The results showed that the clinical skill scores of the intervention group were significantly higher than the baseline levels at 1 week (OSCE2: *P*<.001) and 1 month (OSCE3: *P*<.001) postintervention, suggesting significant short- to medium-term skill-enhancing effects. However, its follow-up study [28] assessed triage skills by chi-square test with OSCE and found no significant difference between the intervention and control groups in the total score of the Triage Skills Questionnaire and scores of the 3 dimensions of Rapid Assessment, Categorization, and Patient Assignment (*P*>.05), suggesting that there may be a scenario-specific limitation in the effect of triage skills migration. In addition, Zhixia et al [39] showed that the total score of trauma scene rescue skill operation of the experimental group was significantly higher than that of the control group (*P*<.05), indicating that the gamification intervention has a facilitating effect on the specific skills.

#### Engagement and Knowledge Retention

Five studies (5/16, 31.3%) assessed engagement through satisfaction surveys, and the results showed that participants generally recognized the interactivity and fun of the gamified teaching format [29-31,34,36]. However, the long-term knowledge retention effect showed heterogeneity: Chang et al [34] found no significant difference in chemical disaster knowledge scores between the intervention and control groups 3 weeks after the simulation game intervention (*P*>.05), whereas Masoumian et al [31] noted that the skill knowledge of the intervention group was still significantly higher than the baseline level at the 1-month follow-up period (*P*<.01), but the theoretical knowledge difference did not reach statistical significance. Such contradictions may be related to the length of the intervention, the form of the technique, and the sensitivity of the assessment tool, and further research is needed to validate them.

#### Self-Efficacy

Two studies indicate that self-efficacy has a significant impact on the learning and use of computers [41]. For example, the study by Chow et al [33] revealed that computer self-efficacy accounted for a substantial portion of the variance in perceived ease of use and perceived usefulness. Furthermore, individuals with higher self-efficacy exhibited elevated expectations for computer use, aligning with motivation theory and Bandura’s social cognitive theory [42]. The research by Chang et al [34] demonstrated that both virtual reality and tabletop exercises could enhance nursing professionals’ self-efficacy in responding to chemical disasters, particularly among less experienced nurses, with virtual reality notably improving their short-term self-preparedness.

#### Quality Evaluation Results

**Figure 2.**
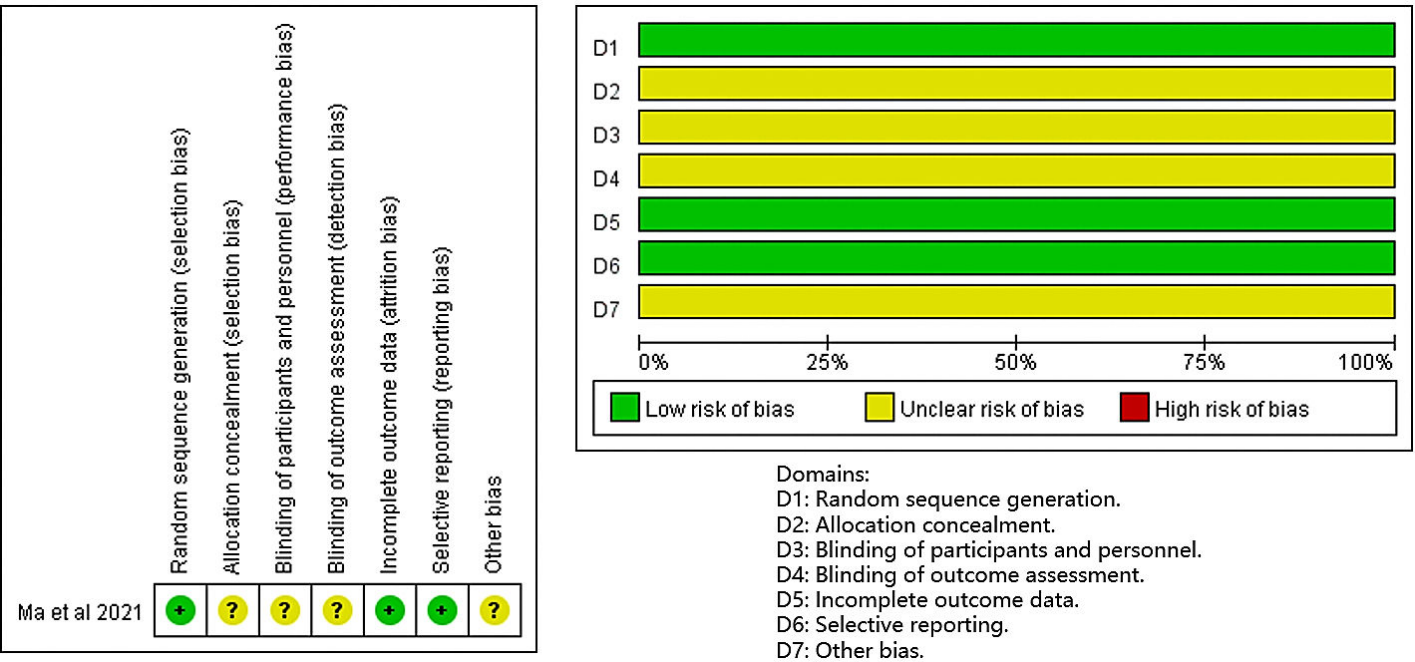
Graph and summary of the risk of bias in the randomized controlled trial studies according to the revised version of the Cochrane risk of bias tool for randomized trials [35].

The methodological quality of the included studies was evaluated using the Cochrane Risk of Bias Assessment Tool and the Joanna Briggs Institute Risk of Bias in Non-Randomized Intervention Studies tool. The overall quality of the studies was found to be average. The results indicated that the sole included RCT was assessed as having a medium risk regarding randomized sequence generation and allocation concealment [35], primarily due to the lack of information on whether the study was blinded for subjects and investigators. Among the 15 quasi-experimental studies conducted by the implementers, 11 were classified as high risk of bias [25-27,29,30,32,33,37-40]. This classification arose mainly from deficiencies in 9 areas, including the absence of diversified measurements for implementation outcomes, lack of a control group, or reliance on self-designed instruments for outcome indicators, which significantly heightened the risk of bias. Ultimately, only 4 of the 16 included studies were rated as low risk of bias [28,31,34,36]. The risk of bias graph and summary are shown in Figures2  3, respectively.

**Figure 3.**
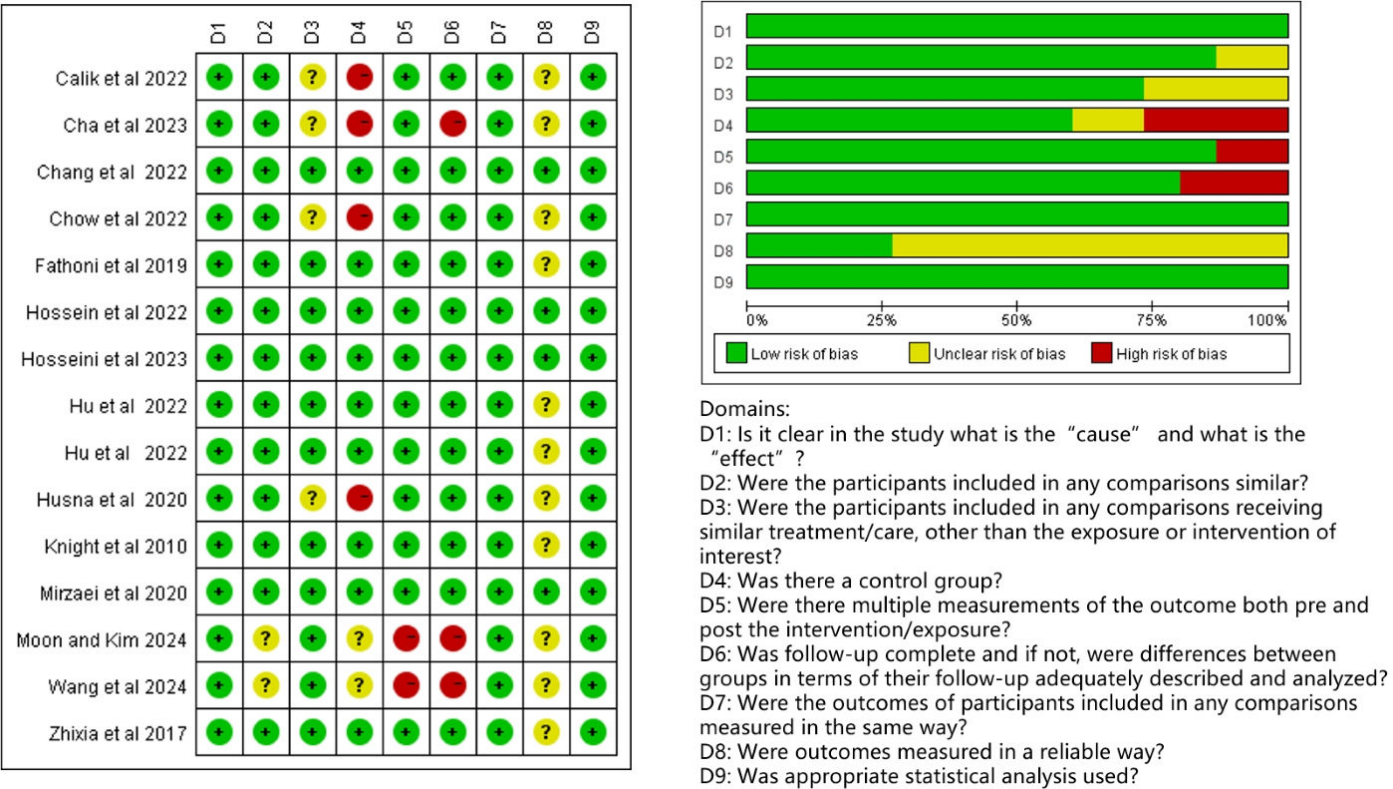
Graph and summary of the risk of bias in the non–randomized controlled trial studies according to the Joanna Briggs Institute risk of bias in nonrandomized studies of interventions [25-34,36-40].

## Discussion

### Principal Findings

The primary objective of this review was to assess the effectiveness of gamified instruction in disaster nursing education and its associated indicators. Although the indicators of effectiveness and the quality of the studies varied, all interventions demonstrated a positive impact on health care workers’ outcomes. Specifically, these interventions significantly improved theoretical knowledge, practical skills, engagement and knowledge retention, satisfaction with the intervention, and self-efficacy.

### Current Status of Gamification

Research has shown that the use of gamified teaching methods in disaster nursing education demonstrates significant results [43,44]. However, gamification differs from digital game design in that it promotes knowledge acquisition and behavioral improvement not only through competition and reward motivation but also through contextual simulation and feedback mechanisms [31]. Tabletop games, for example, cost less than digital games, can simulate situations repeatedly at less cost, and are easy to rehearse, with participants recognizing their roles and responsibilities, which is consistent with previous research findings [26]. Of course, tabletop games have certain limitations. Studies have pointed out that tabletop exercises cannot replace the experience gained from actual disaster response exercises and that learners must have some prior knowledge of disaster response to achieve effective learning outcomes [36]. Therefore, tabletop games are more appropriate for learners with some experience in disaster response, and they are not a particularly effective teaching method for less experienced nurses.

In addition, the core of digital games as complete metadata information systems lies in the systematic description and dynamic interaction of game elements through structured data models, and their design goals are more focused on constructing self-consistent virtual experiences rather than direct educational transformations [45,46]. For example, serious games and 3D video games are also effective in enhancing knowledge and skills. For less experienced nurses, these games are easy to pick up, more engaging, and can increase the cognitive load to some extent [33]. However, these games may also increase extraneous loads, such as prolonged engagement requiring sustained attention and endurance. While limited by device penetration and system stability, they may affect learning outcomes [44]. Therefore, although digital games can provide richer learning experiences, their implementation faces certain technical and resource challenges.

In the future, it is necessary to balance technical complexity with pedagogical use, such as optimizing cognitive load distribution through hierarchical design and developing standardized tools (eg, cross-platform simulation systems) to lower the hardware threshold, to maximize the pervasiveness and effectiveness of gamified interventions in disaster care teaching.

### Outcomes and Effectiveness

Research indicates that the predominant methods for evaluating the effectiveness of gamified instruction involve comparing knowledge and skill differences between intervention and control groups, or assessing changes in knowledge preintervention and postintervention within a single group. Unlike prior reviews of gamified instruction in disaster education [47], the studies included in this review primarily demonstrated the effectiveness of gamified instruction by reporting levels of theoretical knowledge, whereas earlier studies emphasized the availability of game types and outcomes.

This review yields positive conclusions regarding the efficacy of gamified teaching in enhancing the theoretical knowledge and skills of nursing professionals. In 5 studies, standardized questionnaires that were tested for reliability and validity were used to measure theoretical knowledge, thereby ensuring the robustness of the assessment results [28,31,34-36]. However, some studies used self-designed questionnaires, which may complicate the comparison and interpretation of results. Consequently, future research should prioritize the use of standardized, validated assessment tools. Furthermore, 3 studies evaluated skill acquisition through the OSCE, a scientific and objective method that accurately assesses participants’ practical skills in critical areas such as triage, disaster management, and emergency response [28,31,39]. This assessment type is crucial as it enhances the practicality and applicability of disaster nursing education. Additionally, 5 studies indicated that participants generally held a positive attitude toward the gamified teaching format, suggesting that gamified instruction possesses significant advantages in stimulating learning interest and enhancing motivation [26,29,32,33,37]. The promotion of gamified teaching in disaster nursing education is widely accepted and recognized.

However, considerable variation exists in assessment results concerning the long-term effects of gamified instruction, which may arise from several factors. First, differences in intervention duration can influence knowledge retention. For instance, a 5-week instructional intervention that developed an app and incorporated various gamification elements, such as collaboration, storytelling, and competition, resulted in relatively stable knowledge retention 1 month postintervention [31]. In contrast, a study using a brief 2-hour educational game demonstrated a significant decline in knowledge retention 1 month after the intervention [36]. Second, the richness of gamification elements was strongly correlated with knowledge retention outcomes. A singular game model (eg, relying solely on contextual narratives) may diminish long-term effects [36], whereas an instructional design that integrates multidimensional game mechanisms (eg, resource management, time pressure, and immediate feedback) is more conducive to long-term knowledge stabilization [31]. Third, the construction of learning scenarios also impacts knowledge retention. Research indicates that self-directed learning models lacking instructor guidance and external situational support, which primarily depend on stand-alone mobile software, experienced a sustained decline in knowledge retention after 6 weeks [32], underscoring the importance of creating an external learning environment to maintain learning outcomes. Additionally, the heterogeneity of study populations, variations in the reliability of assessment tools, and the diversity of gamified instructional formats (eg, apps, physical games, virtual reality, etc) may further restrict the comparability of results across studies.

### Future Research

Through a systematic review of existing studies, we identified several limitations in the application of gamification methods in disaster nursing education, despite their noted successes. First, regarding research design, the majority of studies used quasi-experimental frameworks, lacking robust evidence from RCTs, and many had small sample sizes, potentially compromising the validity of their findings. Furthermore, significant variability in the implementation of gamified instruction, assessment tools, and training durations across studies has resulted in limited comparability of outcomes [48]. Second, the impact of cultural and educational backgrounds on the effectiveness of gamification warrants consideration. For instance, pedagogical approaches that prove effective in technologically advanced virtual reality settings may not translate well to resource-limited environments [43]. Third, the existing literature has not evaluated cost-effectiveness, hindering the ability to ascertain the relationship between costs and benefits. Finally, while gamified instruction is typically conducted in simulated environments, existing studies have not assessed its clinical performance in real-life disaster scenarios, which are characterized by unique tensions, stresses, and complexities, as well as various uncontrollable factors that may influence performance in disaster care.

In summary, to enhance the scientific and practical value of disaster nursing education research, future studies should focus on optimizing the following aspects: First, it is recommended to establish a standardized assessment framework, uniformly adopting indicators such as knowledge retention rates and mastery of practical skills, along with measurement tools such as the Structured Clinical Assessment Scale. Longitudinal assessments should be conducted at predefined intervals (eg, 1 month and 3 months postintervention) to improve result comparability. Additionally, adaptive gamified instructional programs should be designed based on local cultural contexts and technological resources. Second, research design should prioritize multicenter RCTs and increase sample sizes to enhance external validity. The synergistic effects of gamified teaching and other methods (eg, situational simulation and didactic approaches) should also be explored to construct a diversified educational framework that considers efficiency and population characteristics. Furthermore, studies should examine the differences between gamified teaching and actual clinical performance, particularly in real disaster scenarios, while remaining vigilant to the risks of overimmersion and distraction in virtual environments. Finally, most existing studies focus on short-term effect assessments; while lightweight game elements (eg, points and badges) may motivate participants in the short term, they often fail to promote long-term memory integration due to a lack of in-depth narratives and contextual coherence. Therefore, future studies should further validate the long-term effects of various training modalities.

### Limitations

This study adhered rigorously to the PRISMA framework to ensure methodological integrity. However, several limitations were identified. First, the quality of the original studies was constrained, with 68.75% (11/16) of the included studies exhibiting a high risk of bias. Key issues included the absence of control groups, unvalidated assessment tools, and a singular focus on outcome measures. Second, literature inclusion was biased. Due to the research team’s language proficiency limitations, only Chinese and English literature was considered, excluding gray literature and potentially omitting significant evidence from non–English-speaking regions. Third, the reliability of measurement instruments was questionable, as some studies failed to explicitly detail the tools used for knowledge assessment and their reliability and validity, precluding assumptions of high reliability for the outcome measures. Fourth, sample representativeness was limited; to ensure population homogeneity, studies with mixed health care workforces were excluded, which may diminish the applicability of findings to interprofessional teams. Fifth, there exists a risk of extrapolating findings, as existing evidence predominantly originates from Asian countries, and the empirical application of gamified teaching in disaster care remains nascent, thereby constraining the generalizability of results. Notably, while the stringent inclusion criteria for quasi-experimental and experimental studies enhance the level of evidence, they also reduce the analyzable sample size. Future research should broaden methodological inclusiveness to strengthen the relevant evidence.

### Conclusions

By systematically integrating multidimensional evidence and proposing a classification framework for the first time, this study addresses a significant gap in the field, which has lacked a comprehensive evaluation of effectiveness, and provides clear guidance to educators regarding the selection of gamification tools (eg, virtual reality for simulating high-stakes scenarios and tabletop gaming for resource-constrained environments).

Overall, the systematic review indicates that gamified teaching holds substantial value for both nursing students and registered nurses, enhancing not only disaster care knowledge but also practical skills (eg, triage, evacuation decision-making, and infectious disease management). However, the successful implementation of gamified instruction hinges on the careful selection of target learners, learning content, and suitable gamified elements. Furthermore, future research should explore a hybrid teaching model and develop a unified system of assessment indicators (eg, standardized OSCE scales and long-term follow-up protocols) to mitigate interstudy heterogeneity, enhance result comparability, and facilitate the broader application of gamified teaching in disaster nursing education.

## Supplementary material

10.2196/74955 Multimedia Appendix 1 Search strategy.

10.2196/74955 Checklist 1 PRISMA (Preferred Reporting Items for Systematic reviews and Meta-Analyses) checklist.

## Abbreviations

OSCE: Objective Structured Clinical Examination
PRISMA: Preferred Reporting Items for Systematic Reviews and Meta-Analyses
RCT: randomized controlled trial
