# Supplementary material for: Effectiveness of Gamified Teaching in Disaster Nursing Education for Health Care Workers: Systematic Review
Source: J Med Internet Res. 2025 Jul 9;27:e74955. doi: 10.2196/74955 (PMC12266611; doi:10.2196/74955)
Supplement: Multimedia Appendix 1 [file jmir-v27-e74955-s001.docx]

# **Search Terms Protocol**

**1.PubMed**

| Number | Search Query | Results |
| --- | --- | --- |
| #1 | "Nurses"[Mesh] | 102317 |
| #2 | ((((((Nurse[Title/Abstract])) OR (Personnel, Nursing[Title/Abstract])) OR (Nursing Personnel[Title/Abstract])) OR (Registered Nurses[Title/Abstract])) OR (Nurse Registered[Title/Abstract])) OR (Registered Nurse[Title/Abstract]) | 163854 |
| #3 | "Students, Nursing"[Mesh] | 33129 |
| #4 | (((((((Pupil Nurses[Title/Abstract])) OR (Student, Nursing[Title/Abstract])) OR (Nurses, Pupil[Title/Abstract])) OR (Nurse, Pupil[Title/Abstract])) OR (Pupil Nurse[Title/Abstract])) OR (Nursing Student[Title/Abstract])) OR (Nursing Students[Title/Abstract]) | 24385 |
| #5 | ((((((((((Pupil Nurses[Title/Abstract])) OR (Student, Nursing[Title/Abstract])) OR (Nurses, Pupil[Title/Abstract])) OR (Nurse, Pupil[Title/Abstract])) OR (Pupil Nurse[Title/Abstract])) OR (Nursing Student[Title/Abstract])) OR (Nursing Students[Title/Abstract])) OR (((((((Nurse[Title/Abstract])) OR (Personnel, Nursing[Title/Abstract])) OR (Nursing Personnel[Title/Abstract])) OR (Registered Nurses[Title/Abstract])) OR (Nurse Registered[Title/Abstract])) OR (Registered Nurse[Title/Abstract]))) OR ("Students, Nursing"[Mesh])) OR ("Nurses"[Mesh]) | 260297 |
| #6 | "Disaster Nursing"[Mesh] | 8 |
| #7 | Medicine, Disaster[ Title/Abstract] | 27 |
| #8 | "Disasters"[Mesh] | 116986 |
| #9 | #6 or #7 or #8 | 141388 |
| #9 | ((((((disasters[Title/Abstract])) OR (catastrophe[Title/Abstract])) OR (disaster[Title/Abstract])) OR (Disasters[MeSH Terms])) OR (Medicine, Disaster[Title/Abstract])) OR (Disaster Nursing[MeSH Terms]) | 141388 |
| #10 | ((((((((((((((Gam*[Title/Abstract])) OR (game[Title/Abstract])) OR (Gamification[Title/Abstract])) OR (game-based learning[Title/Abstract])) OR (Educational game[Title/Abstract])) OR (games[Title/Abstract])) OR (learning game [Title/Abstract])) OR (escape room[Title/Abstract])) OR (Serious Games[Title/Abstract])) OR (Video Games[Title/Abstract])) OR (virtual reality[Title/Abstract])) OR (tabletop game*[Title/Abstract])) OR (board game*[Title/Abstract])) OR (simulation game*[Title/Abstract]) | 78770 |
| #11 | #5 and #9 and #10 | 33 |
| #11 | ((((((((((((Pupil Nurses[Title/Abstract])) OR (Student, Nursing[Title/Abstract])) OR (Nurses, Pupil[Title/Abstract])) OR (Nurse, Pupil[Title/Abstract])) OR (Pupil Nurse[Title/Abstract])) OR (Nursing Student[Title/Abstract])) OR (Nursing Students[Title/Abstract])) OR (((((((Nurse[Title/Abstract])) OR (Personnel, Nursing[Title/Abstract])) OR (Nursing Personnel[Title/Abstract])) OR (Registered Nurses[Title/Abstract])) OR (Nurse Registered[Title/Abstract])) OR (Registered Nurse[Title/Abstract]))) OR ("Students, Nursing"[Mesh])) OR ("Nurses"[Mesh])) AND (((((((disasters[Title/Abstract])) OR (catastrophe[Title/Abstract])) OR (disaster[Title/Abstract])) OR (Disasters[MeSH Terms])) OR (Medicine, Disaster[Title/Abstract])) OR (Disaster Nursing[MeSH Terms]))) AND (((((((((((((((Gam*[Title/Abstract])) OR (game[Title/Abstract])) OR (Gamification[Title/Abstract])) OR (game-based learning[Title/Abstract])) OR (Educational game[Title/Abstract])) OR (games[Title/Abstract])) OR (learning game [Title/Abstract])) OR (escape room[Title/Abstract])) OR (Serious Games[Title/Abstract])) OR (Video Games[Title/Abstract])) OR (virtual reality[Title/Abstract])) OR (tabletop game*[Title/Abstract])) OR (board game*[Title/Abstract])) OR (simulation game*[Title/Abstract])) | 33 |

**2.Web of science**

| Number | Search Query | Results |
| --- | --- | --- |
| #1 | (((((((TS=(Nurses)) OR AB=(Nurse)) OR AB=(Personnel, Nursing)) OR AB=(Nursing Personnel)) OR AB=(Registered Nurses)) OR AB=(Nurse Registered)) OR AB=(Nurses Registered)) OR AB=(Registered Nurse) | 1101886 |
| #2 | (((((((TS=(Students, Nursing)) OR AB=(Pupil Nurses)) OR AB=(Student, Nursing)) OR AB=(Nurses, Pupil)) OR AB=(Nurse, Pupil)) OR AB=(Pupil Nurse)) OR AB=(Nursing Student)) OR AB=(Nursing Students) | 96850 |
| #3 | #1OR#2 | 1101866 |
| #4 | ((((TS=(Disaster Nursing)) OR TS=(Disasters)) OR AB=(Medicine, Disaster)) OR AB=(catastrophe)) OR AB=(disaster) | 264717 |
| #5 | ((((((((((((((TS=(Gamification)) OR AB=(Gam*)) OR AB=(Gam*)) OR AB=(Gamification)) OR AB=(game-based learning)) OR AB=(Educational game)) OR AB=(games)) OR AB=(learning game)) OR AB=(escape room)) OR AB=(Serious Games)) OR AB=(Video Games)) OR AB=(virtual reality)) OR AB=(tabletop game*)) OR AB=(board game*)) OR AB=(simulation game*) | 1517921 |
| #6 | #3 AND #4 AND#5 | 79 |

**3.CINAHL**

| Number | Search Query | Results |
| --- | --- | --- |
| #1 | (nurses or nursing staff or nurse) OR (students, nursing or education, nursing) | 641725 |
| #2 | (disaster nursing and emergency preparedness) OR (disasters or natural disasters or tsunamis or floods or drought or wildfire or earthquake or tornado or hurricane or snowstorm) | 40073 |
| #3 | (gamification in education or game-based learning) OR (games in education or game-based learning or educational games) | 1002 |
| #4 | #1 AND #2 AND #3 | 3 |

**4.Embase**

| Number | Search Query | Results |
| --- | --- | --- |
| #1 | 'nurse'/exp OR nurse:ab OR 'personnel, nursing':ti,ab,kw OR 'nursing personnel':ti,ab,kw OR 'registered nurses':ti,ab,kw OR 'nurse registered':ti,ab,kw OR 'nurses registered':ti,ab,kw | 318841 |
| #2 | 'nursing student'/exp OR 'pupil nurses':ti,ab,kw OR 'student, nursing':ti,ab,kw OR 'nurses, pupil':ti,ab,kw OR 'nurse, pupil':ti,ab,kw OR 'pupil nurse':ti,ab,kw OR 'nursing student':ti,ab,kw OR 'nursing students':ti,ab,kw | 41730 |
| #3 | #1 OR #2 | 350994 |
| #4 | 'disaster nursing'/exp OR 'disaster'/exp OR 'medicine, disaster':ti,ab,kw OR 'disasters':ti,ab,kw OR 'disaster':ti,ab,kw | 60960 |
| #5 | 'gamification'/exp OR 'game'/exp OR 'game-based learning':ti,ab,kw OR 'educational game':ti,ab,kw OR games:ti,ab,kw OR 'learning game':ti,ab,kw OR 'escape room':ti,ab,kw OR 'serious games':ti,ab,kw OR 'video games':ti,ab,kw OR 'virtual reality':ti,ab,kw OR 'tabletop game*':ti,ab,kw OR 'board game*':ti,ab,kw OR 'simulation game*':ti,ab,kw | 61891 |
| #6 | #3 AND #4 AND #5 | 23 |

**5.Cochrane Library**

| Number | Search Query | Results |
| --- | --- | --- |
| #1 | MeSH descriptor: [Students, Nursing] explode all trees | 920 |
| #2 | (Pupil Nurses):ti,ab,kw OR (Student, Nursing):ti,ab,kw OR (Nurses, Pupil):ti,ab,kw OR (Pupil Nurse):ti,ab,kw OR (Nursing Student):ti,ab,kw (Word variations have been searched) | 4774 |
| #3 | MeSH descriptor: [Nursing] explode all trees | 4419 |
| #4 | (Personnel, Nursing):ti,ab,kw OR (Nursing Personnel):ti,ab,kw OR (Registered Nurses):ti,ab,kw OR (Nurse Registered):ti,ab,kw OR (Registered Nurse):ti,ab,kw (Word variations have been searched) | 8211 |
| #5 | #1 or #2 or #3 or #4 | 15821 |
| #6 | MeSH descriptor: [Disasters] explode all trees | 1 |
| #7 | MeSH descriptor: [Disaster Nursing] explode all trees | 3769 |
| #8 | (disasters):ti,ab,kw OR (catastrophe):ti,ab,kw OR (disaster):ti,ab,kw OR (Disasters):ti,ab,kw OR (Medicine, Disaster):ti,ab,kw (Word variations have been searched) | 90 |
| #9 | #6 or #7 or #8 | 3769 |
| #10 | MeSH descriptor: [Gamification] explode all trees | 28 |
| #11 | (Gam*):ti,ab,kw OR (game):ti,ab,kw OR (Gamification):ti,ab,kw OR (game-based learning):ti,ab,kw OR (Educational game):ti,ab,kw (Word variations have been searched) | 28617 |
| #12 | #10 or #11 | 28617 |
| #13 | #5 and #9 and #12 | 13 |

**6.SCOPUS**

| Number | Search Query | Results |
| --- | --- | --- |
| #1 | ( TITLE-ABS-KEY ( nurses ) OR TITLE-ABS-KEY ( personnel, AND nursing ) OR TITLE-ABS-KEY ( nursing AND personnel ) OR TITLE-ABS-KEY ( registered AND nurses ) OR TITLE-ABS-KEY ( nurse AND registered ) OR TITLE-ABS-KEY ( nurses AND registered ) OR TITLE-ABS-KEY ( registered AND nurse ) ) OR ( TITLE-ABS-KEY ( students, AND nursing ) OR TITLE-ABS-KEY ( pupil AND nurses ) OR TITLE-ABS-KEY ( student, AND nursing ) OR TITLE-ABS-KEY ( nurses, AND pupil ) OR TITLE-ABS-KEY ( nurse, AND pupil ) OR TITLE-ABS-KEY ( pupil AND nurse ) OR TITLE-ABS-KEY ( nursing AND student ) AND TITLE-ABS-KEY ( nursing AND students ) ) | 604705 |
| #2 | ( TITLE-ABS-KEY ( disasters ) OR TITLE-ABS-KEY ( catastrophe ) OR TITLE-ABS-KEY ( disaster ) OR TITLE-ABS-KEY ( medicine, AND disaster ) ) | 287650 |
| #3 | ( TITLE-ABS-KEY ( gam* ) OR TITLE-ABS-KEY ( game ) OR TITLE-ABS-KEY ( gamification ) OR TITLE-ABS-KEY ( gamification AND in AND education ) OR TITLE-ABS-KEY ( educational AND game ) ) | 1954469 |
|  | #1 AND #2 AND #3 | 35 |
| #4 | ( ( ( TITLE-ABS-KEY ( students, AND nursing ) OR TITLE-ABS-KEY ( pupil AND nurses ) OR TITLE-ABS-KEY ( student, AND nursing ) OR TITLE-ABS-KEY ( nurses, AND pupil ) OR TITLE-ABS-KEY ( nurse, AND pupil ) OR TITLE-ABS-KEY ( pupil AND nurse ) OR TITLE-ABS-KEY ( nursing AND student ) AND TITLE-ABS-KEY ( nursing AND students ) ) ) OR ( ( TITLE-ABS-KEY ( nurses ) OR TITLE-ABS-KEY ( personnel, AND nursing ) OR TITLE-ABS-KEY ( nursing AND personnel ) OR TITLE-ABS-KEY ( registered AND nurses ) OR TITLE-ABS-KEY ( nurse AND registered ) OR TITLE-ABS-KEY ( nurses AND registered ) OR TITLE-ABS-KEY ( registered AND nurse ) ) ) ) AND ( ( TITLE-ABS-KEY ( disasters ) OR TITLE-ABS-KEY ( catastrophe ) OR TITLE-ABS-KEY ( disaster ) OR TITLE-ABS-KEY ( medicine, AND disaster ) ) ) AND ( ( TITLE-ABS-KEY ( gam* ) OR TITLE-ABS-KEY ( game ) OR TITLE-ABS-KEY ( gamification ) OR TITLE-ABS-KEY ( gamification AND in AND education ) OR TITLE-ABS-KEY ( educational AND game ) ) ) | 35 |

**7.CNKI**

| Number | Search Query | Results |
| --- | --- | --- |
| **#**1 | （TKA 护士 + 护生 + 护理学生 + 注册护士 + 临床护士 + 实习护士 + 护理人员 + 实习护生）AND (TKA 灾难 + 灾难护理 +灾难应对 + 灾难救援) AND (TKA 游戏 + 游戏化教学) | 5 |

**8.WanFang**

| Number | Search Query | Results |
| --- | --- | --- |
| **#**1 | 全部:(护士 or 临床护士 or 护理学生 or 护生) and 全部:(灾难 or 灾害 or 灾害护理) and 全部:(游戏 or 游戏教学) | 55 |
